# Psychotherapy in the Framework of Embodied Cognition—Does Interpersonal Synchrony Influence Therapy Success?

**DOI:** 10.3389/fpsyt.2021.562490

**Published:** 2021-03-22

**Authors:** Melinda A. Mende, Hendrikje Schmidt

**Affiliations:** Potsdam Embodied Cognition Group, Division of Cognitive Sciences, Department of Psychology, University of Potsdam, Potsdam, Germany

**Keywords:** psychotherapy, embodied cognition, hyperscanning, motion energy analysis, neurofeedback, EEG, fMRI, fNIRS

## Abstract

Mental health problems remain among the main generators of costs within and beyond the health care system. Psychotherapy, the tool of choice in their treatment, is qualified by social interaction, and cooperation within the therapist-patient-dyad. Research into the factors influencing therapy success to date is neither exhaustive nor conclusive. Among many others, the quality of the relationship between therapist and patient stands out regardless of the followed psychotherapy school. Emerging research points to a connection between interpersonal synchronization within the sessions and therapy outcome. Consequently, it can be considered significant for the shaping of this relationship. The framework of Embodied Cognition assumes bodily and neuronal correlates of thinking. Therefore, the present paper reviews investigations on interpersonal, non-verbal synchrony in two domains: firstly, studies on interpersonal synchrony in psychotherapy are reviewed (synchronization of movement). Secondly, findings on neurological correlates of interpersonal synchrony (assessed with EEG, fMRI, fNIRS) are summarized in a narrative manner. In addition, the question is asked whether interpersonal synchrony can be achieved voluntarily on an individual level. It is concluded that there might be mechanisms which could give more insights into therapy success, but as of yet remain uninvestigated. Further, the framework of embodied cognition applies more to the current body of evidence than classical cognitivist views. Nevertheless, deeper research into interpersonal physical and neurological processes utilizing the framework of Embodied Cognition emerges as a possible route of investigation on the road to lower drop-out rates, improved and quality-controlled therapeutic interventions, thereby significantly reducing healthcare costs.

## Introduction

Psychological psychotherapy is an important method of treatment in modern society. The number of individuals with psychiatric diagnoses constitutes a considerable part of the general population; in Germany, for example, the proportion of individuals with a diagnosed mental health disorder amounts to close to 30%. Interestingly, not all of the afflicted establish contact with available sources of aid, such as psychotherapists [see e.g., DGPPN (1) and Jacobi et al. (2)], but only a mere 20% [see e.g., Mack et al. (3)]. In absolute numbers, we are looking at a population of approximately three million. This number indicates the relevance of (re-) examining and improving therapeutic interventions—particularly when considering that psychiatric disorders take fourth position (following cardiovascular disease, malignant growths, and musculoskeletal disorders) among the chief reasons decreasing individual quality of life, i.e., the years lived in good health (4).

Mental health problems coincide with a relative mental inflexibility in the afflicted [e.g., (5–7)]. One instance in which this inflexibility becomes apparent is social interaction; in particular, the ability to engage in and uphold non-verbal interaction appears to be diminished by psychiatric disorders [e.g., (8, 9)]. Simultaneously, we experience interaction with others as easier whenever our counterpart adjusts their behavior to match ours (10). Psychotherapy is a multimodal practice with the goal to understand and offer strategical support to patients attempting to manage their problems; as such, its effectiveness is mediated by a myriad of factors [e.g., (11)]. Psychotherapy is one of the tools used by modern medicine to alleviate the suffering caused by ailments such as depression, anxiety disorders, attention deficit hyperactivity disorder (ADHD) or schizophrenia [e.g., (12–16)].

Individual psychotherapy posits a dyadic situation, which can be understood in terms of a leader-follower-paradigm, meaning that one of the two individuals present leads the other. In a therapeutic setting, the assignment of the leader- and follower role can take place both ex- and implicitly. Patients' impaired mental flexibility may contribute to their difficulties in adapting to a given situation, including the therapeutic one [e.g., (17)]. To date, research on the parameters influencing the success of psychotherapy for psychiatric disorders remains far from conclusive. Among the possible variables exerting an influence the relationship quality between therapist and patient seem to outstand all others regardless of the followed therapeutical direction (18, 19).

### Interpersonal Dynamics in Psychotherapy

All major frameworks in psychotherapy took the interpersonal dynamics within the therapeutic dyad into account. The following abstract briefly introduces different theories covered by cognitive behavioral, psychodynamic and humanistic psychotherapy without being able to go into the full depths of the rich theoretical and empirical backgrounds.

Firstly, for the description of interpersonal processes in cognitive behavioral therapy (CBT), Safran (20) refers to the interpersonal theory by (21) and introduces the concept of interpersonal schema to integrate cognitive and interpersonal factors into CBT. Those schemas are defined as “generic cognitive representations of interpersonal events” (20). Following the ecological approach by Gibson (22) individuals act within the context of their environment in a functional way. An important part of those survival relevant interactions within the real world contains interpersonal interactions and is related to rather ancient structures within the human brain for social and emotional processes (23). Interpersonal schemas are activated to maintain interpersonally related. Distinctive cognitive-interpersonal cycles contain cognitive processes that lead to behaviour and communication and consequently validate specific interpersonal schemas (24). Within the relationship between psychotherapist and patient such self-other-relationship representations are salient on both sides and can be integrated into the therapy process by the therapist in a constructive way. Compassion-focussed therapy (CFT) emphasizes the important role of the motivational system activated within interpersonal relatedness that enables caring, helping and sharing emotions and behavior (23).

Secondly, within psychodynamic and psychoanalytical theory and practice, the representation of objects' relations and mentalisation of others is highly emphasized. Freud defined the object (i.e., other people or parts of them) as the source and driver of activation of the individual—the subject. The object's representation evokes a certain emotion within the subject. The relationship to the therapist is directly addressed within psychodynamic therapy by reflecting the patients' ability to mentalise the mind of another person, in this case the therapist (25). Transference (i.e., the patient's transported emotions and wishes in the therapy process) and countertransference (i.e., the therapist's transported emotions and wishes the therapy process) are used to reveal interpersonal dynamics in the therapy process (26). Cognitive analytic therapy is integrating the object relation theory and constructs of social development associated with CBT. It focusses on the specific patterns of a person in relating to others, so called reciprocal roles (27).

Finally, a truly empathic therapeutic relationship is the core element of humanistic therapy. Schneider and Längle (28) define humanism in psychotherapy as putting “special emphasis on the personal, interpersonal, and contextual dimensions of therapy and on clients' reflections on their relationship with self, others, and the larger psychosocial world”. Person- or client-centered psychotherapy (29) can be seen as the root for further developed humanistic psychotherapy approaches, like emotion-focussed therapy where emotion is the key to self-understanding and construction (30). The interpersonal experience of the client within therapy shall be exclusively emotionally validating. The quality of the relationship offered by the humanistic therapist, which is defined by empathy and appreciation, is perceived as crucial by the clients as it enables them to a deeper understanding of themselves (31).

In light of the above-described approaches, psychotherapists are trained to be aware of and monitor their own as well as the patient's non-verbal behavior (32). This aspect has, over time, received less and less attention, since empirical evidence—according to the Handbook of Psychotherapy and Behavior—has remained sparse (33). But as technology advances, it offers new approaches to investigate non-verbal behavior in the framework of clinical and social psychology (34, 35).

The importance of role-attribution becomes obvious in the study of social settings. For example, Chang et al. (36) were able to show how the cooperation of musicians is influenced by conscious role-assignment. Players in violin quartets exerted a stronger influence on their teammates after being consciously assigned the leader-role. Additionally, they were less prone to have their movements influenced by others than those individuals who spontaneously (i.e., unconsciously) took the leader-role. Interpersonal synchrony may thus improve performance in the cooperation of multiple individuals. Bodily non-verbal synchrony appears to be a valid measurement for social interaction—because it correlates with brain mechanisms (37).

In a recent review, Pan and Cheng (38) emphasized that two-person approaches are useful for assessing social interactions in the setting of psychotherapy—both for interventions and diagnosis. This review provides an extension by discussing neuroscientific studies on subconscious brain synchrony as well as voluntary brain synchronization in a narrative manner.

### The Framework of Embodied Cognition

Traditional philosophical concepts claim that cognition is caused of and affected by brain mechanisms only. Among a variety of classical concepts of mental representation rather amodal representations are assumed. This view has been challenged in the last decades.

One example of the classical views is the theory of dualism, grounded by René Descartes. The main idea of dualism is that the mind and the body are seen as two completely separate entities, so that mental experiences are not physical (39). This concept has been put into question. Accordingly, mechanisms of the body also play a role in cognitive processes, because the mind develops in the brain and experiences made by the body form the mind (40). For instance, Searle (41) illustrated in his Chinese Room example that classical theories of knowledge representation have some shortcomings because applying systematic symbol manipulation only does not lead to comprehension of symbols. Thus, it appears that mental representations are built by experiences made by the body and therefore are embodied. Among all of the classical views about the relationship of mind and body, dualism is the most controversial theory compared to the theory of embodiment.

Converse to the classic cognitivist approach, the Embodied Cognition Theory posits thinking (i.e., representing) to be multimodally connected with the body, thought processes thus being understood as “embodied” (42). This “embodiment” concerns many domains of human existence, such as perceptual processes [e.g., visual perception, (43)], emotional concepts (44), memory retrieval (43), or the interplay of attention, perception, and action (45).

There are many factors that possibly influence human cognition such as cultural factors, social and contextual factors. More recently, it has been considered that habitual factors could also play a role in human cognition (46). Cultural, social and contextual factors are outside of the body, but can have an influence on the feelings of a person, for instance. Habitual factors come from inside the human body such as a movement of a limb into a certain direction. This division is not trivial, because it can help to gain a deeper understanding of the underlying processes of human cognition and learning.

Human individuals are biological beings; the human experience and existence is inextricably entwined with the body which enables it. Thus, psychological and psychiatric phenomena originate in this same body, in particular, the brain. However, psychological issues are far too complex to warrant simplistic explanations; more often than not, they cannot be fully accounted for by a single cause such as a disturbance of the endocrinological system. Psychological impairments can in fact be localized in the brain; however, neuronal processes alone do not constitute what is commonly referred to as “the human mind,” from which psychological phenomena arise. Rather, the latter are formed and shaped by the complex interaction of neuronal processes taking place in—as well as the physiology of—the brain on the one hand, and psychologically acquired patterns, learning processes, and cultural norms on the other (47).

## Synchronization in Human Non-Verbal Communication

A systematic search was conducted to identify relevant literature. The search covered the following databases: Google Scholar, Science Direct, PubMed, PsychInfo, and Web of Knowledge. The following keywords were used in the search “non-verbal synchronization psychotherapy embodied,” “non-verbal synchronization embodiment psychotherapy,” “non-verbal synchrony psychological counseling embodied.” In addition, studies were selected by hand and cited studies in the identified papers were inspected. Evidence is summarized in the following.

In line with the above-mentioned theories of interpersonal dynamics in psychotherapy numerous studies point toward the therapeutic alliance as a key factor in and predictor of a given therapy's success (18, 19). This alliance between both protagonists of an individual psychotherapy (i.e., the therapist and the patient) is considered to be influenced by components such as the shared pursuit of a common goal (e.g., an improvement of the patient's quality of life) and interindividual sympathy, which in turn enable the patient's capacity to change (e.g., their behavior, cognitive and perceptual strategies, and patterns, etc.) (48).

Similarly, therapy success appears to correlate with movement synchrony between therapists and patients, which has been measured via motion energy analysis (MEA) [e.g., (49)]. Since a condition for the successful occurrence of interpersonal synchrony is an at least partial access to the internal states of the other person (50), and this access is key to a functioning therapeutic alliance, both therapy success and therapy drop-out have been successfully predicted by measurements of non-verbal synchrony. Higher measurements of synchrony between therapist and patient at the beginning of therapy go along with a higher rate of amicable (i.e., concerted, mutual) termination of the therapy after its agreed-upon duration. Conversely, synchrony measures were lowest for those patients who chose to terminate their therapy at an early stage (51). This points to the influence of non-verbal synchrony between patient and therapist on the duration of therapy. Additionally, the relative role allocation between both parties plays decidedly influences therapy success: if patients inhabit a leading role during the first three sessions, it increases the chance of an early therapy drop-out (52).

## Other Research on Interpersonal Synchrony

A systematic search was conducted to identify relevant literature. The search covered the following databases: Google Scholar, Science Direct, PubMed, PsychInfo, and Web of Knowledge. The following keywords were used in the search ‘social interaction brain synchrony', ‘social interaction neurofeedback', ‘hyperbrain networks social interaction'. In addition, studies were selected by hand and cited studies in the identified papers were inspected. Evidence is summarized in the following.

Social neuroscience is a comparatively young area of research, which has developed in recent years along with technology and its scientific application. Insights gleaned from single-recording studies of individual research subjects are now enhanced via simultaneous recording of two or more subjects, opening the possibility to measure neural processes of social interaction in real-time [e.g., (37, 53, 54)]. As this new string of research provides new potentials of investigating social interactions in general and non-verbal synchronization in particular, the following paragraph provides a short, narrative overview of studies on interpersonal synchrony in different domains^1^.

### Evidence From Hyperscanning Studies Using Leader-Follower Paradigms

A number of theories have suggested neural synchronization between human individuals as the basis for interpersonal bonding [compare e.g., (55)]. Team performance appears to increase if neural synchrony is present among team members; successful cooperation seems to correlate positively with interpersonal synchrony [e.g., (56)]. Additionally, interpersonal synchrony appears to influence self-esteem [e.g., (57)].

From a technical point of view, studies of interpersonal neural synchronization have already been realized with different methods of measurement, i.e., electroencephalography [EEG; e.g., (37, 54, 58)], magneto-encephalography [MEG, (59)], functional magneto-resonance imaging [fMRI; e.g., (10)], and functional near-infrared spectroscopy [fNIRS; e.g., (60)].

Studies on neural synchrony in social interaction reveal the coupling of brainwaves in people working together, meaning that interacting individuals' brains build a kind of hyperbrain network in different social setups, such as joint music playing (61), romantic kissing (58), interaction of parents and children (62), and team interaction (56). The amount of synchrony of both body and neural correlates in the brain are linked, meaning that following a successful social interaction (e.g., joint finger-tapping), neural synchrony is present in the brains of participants. This emphasizes the reciprocal relationship between physical and neural synchrony (37).

The assignment of leader- vs. follower-role further modulates this relationship. Individuals who intrinsically tend to take a leading position will, when placed in an interaction with a virtual partner (e.g., a metronome), estimate the success of synchronization as higher whenever they are indeed leading. Additionally, fMRI correlates have shown these “natural leaders” to have increased activity in right lateral and frontal areas responsible for cognitive control and self-related processing (10). These findings are easily reconcilable with the observation that the differential roles of leader and follower evoke different strategies of neural processing. For example, Zhou et al. (59), using MEG to measure neural synchronization during a hand-movement-synchronizations-task, found followers to exhibit a higher modulation of beta-waves in the occipital cortex than leaders. This points to a differential processing of kinematic-related visual information, which reflects the control of one's own movements. Comparable results were obtained in an fNIRS study by Vanzella et al. (63) investigating violinists: They, too, observed different neuronal activation patterns in followers when playing a solo (control condition) compared to following another person playing (assigned follower condition), i.e., greater activation of temporo-parietal areas (processing of dynamic social information) and somatomotor areas (motor simulation). However, no such difference was observed in the assigned leader condition, indicating that specifically following increases activity in areas of the brain associated with motor information and dynamic social information. Leading, on the other hand, comes with its own unique activation pattern (54), and feels good: Sabu et al. (64) showed that participants' becoming aware of their leader position in a social interaction was accompanied by increased activity of the caudate nucleus, a brain area related to feelings of reward.

Lastly, an increased interpersonal neural interaction goes along with the circumstance of a leader-follower-relation per sé—meaning that participants adapt naturally to one of these two roles [compared to two individuals trying to lead, resulting in lower overall synchronization; see Jiang et al. (60)]. Further, social interaction has been shown to strengthen the interbrain networks of the participating individuals. A recent study of resting state fNIRS by Zheng et al. (55) observed neural networks and increased affiliative bonding between teachers and students only after lessons in which they took turns explaining the study material, while no such networks were observed after lessons in which only the teacher lectured, or video lectures. The authors interpreted their findings to show that social bonding is modulated by social interaction, an assumption further supported by observations of brain mechanisms such as phase locking, as well as both within- and between-brain coherence support coordinated social interaction (61, 65).

### Is It Possible to Voluntarily Synchronize?

To incorporate conclusions drawn from currently available empirical research into interpersonal action coordination, it appears sensible to investigate whether individuals are able to voluntarily influence their degree of synchronization. Most studies employ specific tasks, e.g., guitar playing [e.g., (61, 65); discussing, (60)]. Other studies have tried to elicit interpersonal coordination without explicit tasks requiring movement or speech; instead, the interpersonal synchronization was visualized using neurofeedback [NFB; (66, 67)].

Neurofeedback (NFB) is a method by which an individual can learn to adapt their own brain oscillations voluntarily and can be realized using EEG (68), fMRI (69), or fNIRS (70). Participants learn to selectively enhance and/or suppress certain frequencies of their own brain waves; in healthy subjects, it can be applied as a means of prevention by training particular skills [e.g., enhancement of the capacity to remember (71)]. Additionally, NFB is a tool applied in various therapies. Effects are usually observed after ~10–12 sessions [e.g., (72, 73)]. Examples of areas of application are treatment of ADHD in children [e.g., (70, 74)], major depressive disorder [e.g., (75, 76)], anxiety disorders and phobias [e.g., (77, 78)], and tinnitus [e.g., (79, 80)], all of which utilize NFB to train voluntary control of brain oscillations. All of these studies employed individual brain computer interfaces (BCIs), meaning that one participant's brain oscillations were projected onto a computer screen.

Taking the aforementioned into account, is it conceivable to replace overt tasks with visual NFB in hyperscanning studies? The literature approaching this question is scarce; and so far, very few studies have assessed the combination of NFB and hyperscanning to investigate social interaction. Namely, these studies applied BCIs on two or more individuals simultaneously, so the real-time NFB of all participants could be calculated without temporal delay. Duan et al. (66) trained participants to voluntarily adapt their brain waves via playing a tug-of-war game against each other (participants had to imagine motor activity). Interestingly, they learned to control their own brain waves only after one training session using fNIRS. Though the training was successful, evidence remains preliminary; but this study shows that it is possible to implement a platform for calculating a visualizing fNIRS NFB in social interaction online. Duan's et al. approach provides new insights into ongoing processes of neural correlates in social interaction. Of particular importance to our question, this paradigm is free of interference with physical motor activity, as participants only imagine movement, but do not actually move.

Another study utilized EEG NFB, testing 20 subjects outfitted with mobile EEG headsets in a natural environment (a carnival) in 4 groups of 5 subjects each. The researchers' goal was to create a test situation as natural as possible; the participants learned to voluntarily manipulate their mental states with regard to relaxation vs. concentration. Visual NFB was provided for alpha and beta frequency ranges, and indeed, a training effect was observed (67). This shows the possibility to voluntarily adapt brainwaves to multiple individuals with the help of collective NFB—not only in a laboratory, but in natural environments.

We have presented methods by which interpersonal synchronization can be measured, differentiation between physical bodily and neural synchrony. From the present evidence, a number of possible conclusions with regards to psychotherapy offer themselves. On the one hand, individuals with mental health issues exhibit a high degree of mental inflexibility. On the other hand, it has been shown that the role (with regards to leader vs. follower) taken by either therapist or patient within the first sessions of psychotherapy is indicative of the therapy's success, likely because it pre-sets the stage on which interaction can take place and alliance can emerge.

## Discussion

Studies of leader-follower behavior measuring neural processes have shown that inhabiting either role leads to the recruitment of additional brain areas. Is there a connection between the ability to recruit these areas and the ability to adapt to others? Here, too, we must ask the question of volition—for an inability to refrain from adaption, i.e., an inability to choose the leader or follower position depending on the situation, is by no means “healthier” than an inability to adapt. Mental health is, among other things, qualified by mental flexibility. That means that psychological health presents with impairment or limitation in mental flexibility. Productive social interaction requires us to adapt to others while maintaining a position from which we do so. If we view the therapeutic setting as a leader-follower paradigm, we may well observe valid predictors for the success of therapeutic interventions. We may also learn valuable lessons with regards to the question if—and if so, how much—neural synchrony plays a part in successful therapeutic treatment. For investigation of the physical synchrony between therapist and patient has already revealed a connection between synchrony, bonding and therapeutic success; as well as that between neural correlates and physical synchro and/or cooperation. So, the question remains whether a measurement of neural synchrony may be a valid predictor of therapy success; and if so, at what point in the timeline of a given therapy this synchrony is crucial.

Yet another question posited by the existing literature is that of the ability to voluntarily achieve neural synchrony with another person. Studies of single participants using BCI have shown humans' principal ability to influence their own brainwaves on different bandwidth, as evidenced by the successful treatment of various mental disorders (e.g., MD, ADHD, anxiety) with NFB. The studies by Duan et al. (66) and Kovacevic et al. (67) have shown that it is technologically possible to combine NFB and hyperscanning. Testing interpersonal neural synchronization via NFB rather than through an explicit physical task allows researchers to disentangle the effects of bodily and neural synchrony, because participants don't have to move. Thereby, NFB studies make it possible to exclude bodily rhythms (generated by e.g., playing guitar or finger-tapping), which are known to influence brain oscillations. Additionally, such studies allow for a valid investigation into the question whether volition and visual feedback alone are enough to facilitate an adaption to another individual's brain oscillation—since it has been shown that the same is true for influencing one's own brainwaves via single person NFB and BCI. Applied to individual therapy, such measurements may well reveal further correlates of (un-) successful therapeutic interaction. Especially interesting would be whether patients would be able to adapt to another person in this situation—would the effects observed with BCI (i.e., an ability to manipulate one's own brainwaves) be transferrable, or would social interaction itself hinder patients' ability to adapt to their therapist?

The therapeutic relationship is valued as crucial across all major psychotherapy schools. Nevertheless, specific impact factors remain rather unclear—synchrony was outlined as one. Carey et al. (81) try to explain the therapeutic effect of the therapeutic relationship by referring to Perception Control Theory (PCT). Following PCT, psychological distress arises from conflicted goals of the patient or client. Within therapy sessions those conflicts shall be resolved. The therapist's agenda in the session should be to provide a warm, trustful and empathic surrounding which enables the patient or client to not only speak openly about their problems but also to reflect on their inner motives. Consequently, the definite goal of psychotherapy, the reduction of psychological distress of the patient, can be achieved (81). The reduction of psychological distress will in turn improve mental flexibility. Patients themselves rule out the perceived therapeutical alliance as major contributor to a successful therapy session. On the contrary, whenever there is perceived conflict of interest or goals between the two involved individuals, therapy process might be slowed down or even stopped. Therefore, the therapist is encouraged to monitor their own goals and adjust them within the therapeutical agenda. Mental flexibility of the patient can be encouraged by the therapist by active conflict solving whenever problems within the therapeutic relationship occur (81). Increasing synchrony between therapist and patient can facilitate the above-described interpersonal dynamics.

Even though there is evidence that synchrony of body movement correlates with perceived therapy success as well as leader-follower assignment. To this date, it is difficult to directly relate the account of embodied cognition to improvement of therapy success. Psychotherapeutic sessions differ from laboratory settings which makes the assessment and use of synchrony measurements difficult. This is especially true for neurological measurements. Therefore, evidence of neuronal synchrony between therapists and patient are not available so far.

On the other hand, neurofeedback is already being applied and established in some fields of psychotherapy such as ADHD [see for example Enriquez-Geppert et al. (82), Marx et al. (70), and van Doren et al. (74)] and schizophrenia [see for example Gandara et al. (83) and Surmeli et al. (84)]. Future research will show whether the framework of embodied cognition and its signatures both in the body and in the brain can help to improve psychotherapy. From this timepoint, it is not common to implement neurological measures such as EEG, fMRI and fNIRS into psychotherapeutic settings. But as technology improves, portable devices, for instance portable EEG-caps are available which allow measuring brain activity in more natural and interactive settings. Therefore, future research on how social cognition in social, therapeutic interaction is reflected in the body becomes probable.

Moreover, research with MEA demonstrates already to today that subconscious synchronization of the body can be utilized to explain phenomena such as therapy drop-out. Future studies on these interrelations might reveal further aspects that influence on the success of psychotherapy.

## Conclusion

Many studies to date have shown interbrain synchronization to be a foundation of social interaction, and to correlate with the effort and/or success of social interaction. The embodied aspects of social interaction offer a window to new insights within the therapeutic setting, as well as to the verification of so far theoretical assumptions about the significance of non-verbal communication and role-assignment between therapist and patient. Not only do we see possibilities for an early assessment of therapy-success-probability; indeed, the investigation of the connection between non-verbal bodily and neural synchrony may widen and deepen our understanding of what it means to “relate,” and how both the body and our intention to approximate another's perspective can help us to do just that. Last but not least, as our understanding of the necessity to flexibly inhabit leading and following positions for successful social interaction grows, we approach yet another quality (namely: role flexibility) that constitutes mental health—and may glean new therapeutic interventions to train just that ability. For a unifying characteristic of mental illnesses is that internal (often unconscious and implicit) presumptions and cognitive processing patterns drive the afflicted individual to behave inflexibly across a number of situations, and that this inflexibility greatly impairs the individual's capacity to form and uphold reciprocally satisfying social interactions. Since social binding is one of the main resources in the maintenance of mental health (by means of reducing stress, increasing self-esteem and offering the possibility for rewarding experiences), it is obvious why the investigation of underlying neural mechanisms and the influence we can exert over them is a worthy one.

As mentioned before, the leader-follower-patterns emerging at the beginning of therapy hint at the probability of durable mutual cooperation and successful treatment. The possibility to measure the interplay of unconscious physical and neural synchrony, as well as the ability to voluntarily enhance this synchrony with regards to another person, opens up a new and promising line of research. NFB has already been evidenced to work in individually treating patients. With new technologies such as fNIRS and portable EEG at hand, we can investigate the neural correlates of therapy success via hyperbrain NFB environments, while simultaneously learning more about the neural processes involved in social cooperation and adaptation.

Nevertheless, the current body of evidence points to the fact that the framework of embodied cognition holds, and that body and mind cannot be separated in two independent entities like the theory of dualism or other classical views suggested. To this point, no direct evidence exists for a specific module in the body or the brain which modifies social interaction. Rather, correlations of brain synchrony can be observed and point toward the fact that interpersonal neural synchrony plays a role in social interaction—also in psychotherapy.

Since psychological disorders are widespread and generate significant costs for governments by drawing on funds dedicated to health care, unemployment insurance and retirement, an improvement of psychotherapy success rates is also of interest from and economic point of view. Raising the chances of healing, lowering drop-out rates and thereby improving a given population's attitude toward psychotherapy would thus lighten the economic burden imposed by mental illness. The rather new technologies of hyperbrain scanning and neurofeedback introduced in this paper offer us valuable future tools to empirically investigate the factors contributing to therapy success. Incorporating the framework of Embodied Cognition—combined with new technologies continually solidifying the same—into the striving for better psychotherapeutic treatment is, in the face of many remaining unknowns, nothing short of a sensible decision.

## Author Contributions

MM: research of discussed studies and writing. HS: research of background and writing.

## Conflict of Interest

The authors declare that the research was conducted in the absence of any commercial or financial relationships that could be construed as a potential conflict of interest.

## Footnotes

^1^see Pan and Cheng (38) for another detailed review on two-person approaches for assessing social interactions in the setting of psychotherapy, where also manipulation of interbrain synchrony is assessed.
